# Supplementary material for: The causal role of circulating inflammatory markers in osteoporosis: a bidirectional Mendelian randomized study
Source: Front Immunol. 2024 Jul 18;15:1412298. doi: 10.3389/fimmu.2024.1412298 (PMC11291241; doi:10.3389/fimmu.2024.1412298)
Supplement: Supplementary file 1 [file DataSheet_1.zip › supplementary material/OSTEOPOROSIS-CIM/GCST90274787.tsv.gz_forest.pdf]

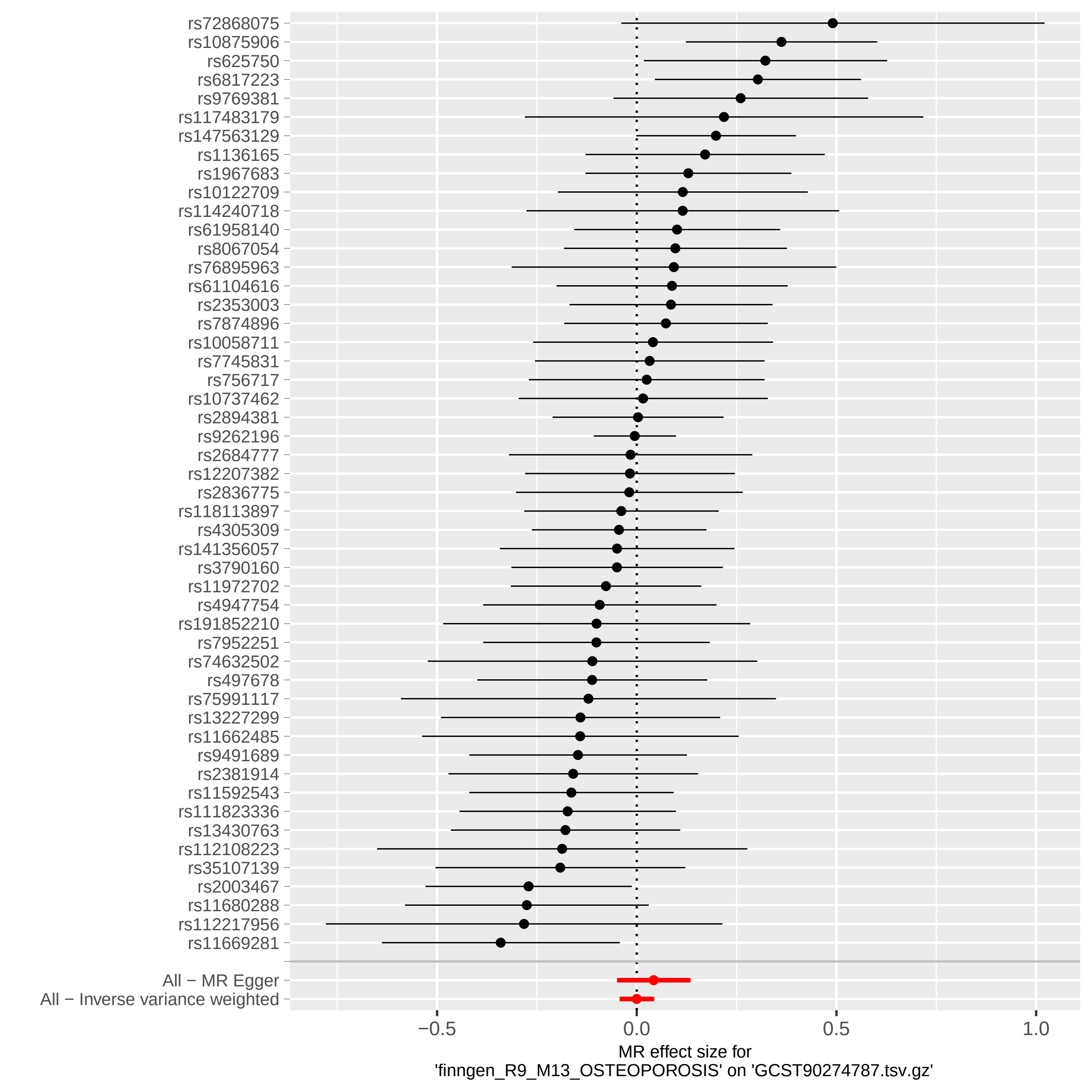
rs72868075
rs10875906
rs625750
rs6817223
rs9769381
rs117483179
rs147563129
rs1136165
rs1967683
rs10122709
rs114240718
rs61958140
rs8067054
rs76895963
rs61104616
rs2353003
rs7874896
rs10058711
rs7745831
rs756717
rs10737462
rs2894381
rs9262196
rs2684777
rs12207382
rs2836775
rs118113897
rs4305309
rs141356057
rs3790160
rs11972702
rs4947754
rs191852210
rs7952251
rs74632502
rs497678
rs75991117
rs13227299
rs11662485
rs9491689
rs2381914
rs11592543
rs111823336
rs13430763
rs112108223
rs35107139
rs2003467
rs11680288
rs112217956
rs11669281
All − MR Egger
All − Inverse variance weighted
−0.5
0.0
0.5
1.0
MR effect size for
'finngen_R9_M13_OSTEOPOROSIS' on 'GCST90274787.tsv.gz'
